# Clinical outcomes of conjunctivochalasis treatment with a new ophthalmic radiofrequency device

**DOI:** 10.1186/s12886-024-03499-2

**Published:** 2024-07-22

**Authors:** Bokyung Kim, Yongwoo Lee, Hyeck-Soo Son, Chul Young Choi

**Affiliations:** 1 Department of Ophthalmology, Kangbuk Samsung Hospital, Sungkyunkwan University School of Medicine, Seoul, Republic of Korea; 2 Department of Ophthalmology, Kangwon National University Hospital, Kangwon National University School of Medicine, Chuncheon, Republic of Korea; 3 The David J. Apple International Laboratory for Ocular Pathology and International Vision Correction Research Centre (IVCRC), Department of Ophthalmology, University of Heidelberg, Heidelberg, Germany

**Keywords:** Conjunctivochalasis, High frequency radiowave, Ophthalmic radiofrequency, Chemosis

## Abstract

**Purpose:**

To investigate the safety and efficacy of a new micro-controlled radiofrequency device for treatment of conjunctivochalasis (Cch).

**Methods:**

Data of 127 patients (230 eyes) who underwent ophthalmic radiofrequency treatment for Cch from January 2020 to June 2023 were analyzed retrospectively. Cch coagulation was performed with a radiofrequency electrode tip (OcuRF®, Ilooda, Korea) and a high-frequency radio-wave electric unit (0.6 ~ 0.8 watts, 2 MHz, Acutron™, Ilooda, Korea). Pre- and postoperative Cch grading, slit-lamp photography, tear film break-up time (TBUT), and bulbar conjunctival hyperemia using Keratograph 5 M (Oculus, Wetzlar, Germany) were evaluated. Cch grade 0 or 1 after surgery was regarded as ‘success’. Complications, recurrence, and additional treatment rates were analyzed.

**Results:**

In 227 (98.7%) eyes, the radiofrequency treatment led to marked improvement of Cch, with 224 (97.4%) eyes achieving grade 0 or 1 at 2 months postoperatively. Eight eyes (3.5%) received additional treatment. TBUT improved from 3.17 ± 0.82 s to 5.28 ± 1.10 s after surgery (*P* < 0.001). The total bulbar conjunctival hyperemia value showed an improvement from 1.7 ± 0.6 to 1.4 ± 0.6 postoperatively (*P* < 0.05). No serious complications were observed.

**Conclusion:**

The novel ophthalmic radiofrequency device led to a marked improvement of Cch with no serious adverse events during the entire follow-up period. Our results suggest that the radiofrequency device presents a safe and efficacious treatment option for Cch.

**Supplementary Information:**

The online version contains supplementary material available at 10.1186/s12886-024-03499-2.

## Introduction

Conjunctivochalasis (Cch) refers to an ocular condition characterized by redundant, stretched conjunctival tissue between the globe and lower eyelid [1]. Reported causes of Cch include age-related elastotic degeneration, conjunctival inflammation, and mechanical friction [2–4], and it often leads to tear-film instability and delayed tear clearance causing dry eye symptoms such as ocular discomfort and epiphora [1, 5–9].

First-line treatment for Cch consists of conservative management including topical lubricants such as artificial tears, anti-inflammatory and antihistamine eye drops, or bandage contact lenses [5, 10]. Patients with severe Cch are often not treated favorably with medical treatment and require surgical treatment. Current mainstay of surgical treatment includes surgical excision, conjunctival cauterization, scleral fixation suture, and radiowave electrosurgery. Following surgical excision, suture, fibrin glue, amniotic membrane transplantation, and tissue grafting can be performed [10]. Despite several treatment options available, consensus on the treatment of choice has not been established for Cch yet.

Previous studies have shown that high-frequency radiowave electrosurgery yields favorable outcomes in reducing Cch, alleviating subjective symptoms, and improving dry eye parameters [11–13]. High frequency devices are already being widely used as safe and effective tools in various medical fields including ophthalmology. Its main advantage lies in its ability to achieve therapeutic effects with minimal damage to surrounding tissue compared to conventional cautery, which utilizes low-frequency and powerful burns that may spread to adjacent tissue [14]. High-frequency radiowave electrosurgery has also been found to allow shorter operation time, faster recovery, fewer complications, and reduced postoperative discomfort than conventional cauterization [11, 12].

The recently Conformity Europe- (CE-) and Food and Drug Administration (FDA)-approved, new micro-controlled radiofrequency treatment device for ophthalmic usage (Acutron™, Ilooda, Korea) comes with 0.2 to 2 watts energy range and an electrode (OcuRF®, Ilooda, Korea) that features a thickness of mere one micron at its tip. To date, however, no reports exist on clinical outcomes of Cch treatment using this device. Herein, the aim of this study was to investigate the clinical outcomes after the application of ophthalmic radiofrequency treatment for Cch.

## Materials and methods

### Subjects

This retrospective study was conducted in accordance with the tenets of the Declaration of Helsinki. It was approved by the Institutional Review Board and Ethics Committee of Kangbuk Samsung Hospital in Seoul, South Korea (IRB No. 2023-06-046-002).

We reviewed medical records of patients who visited the ophthalmology department of Kangbuk Samsung Hospital from January 2020 to June 2023 with a diagnosis of Cch, conjunctival chemosis, nevus, cyst, trichiasis, dry eye, or superior limbic keratitis and underwent ophthalmic micro-radiofrequency procedure.

All patients underwent visual acuity and intraocular pressure measurements, slit lamp examination, anterior slit photography (BQ-900 LED, Haag-streit diagnostics, Mason, OH, USA), and fluorescein staining prior to surgery. Cch was diagnosed when loose conjunctival folds of the inferior lid margin were observed under slit-lamp biomicroscopy. Cch was graded according to Meller and Tseng [5] both before and after surgery. Patients were classified as grade 0 (no persistent fold), grade 1 (one or small fold), grade 2 (≥ 2 folds, but not higher than the tear meniscus), or grade 3 (multiple folds and higher than the tear meniscus). Patients with Cch grade of 2 or higher were considered eligible for surgical treatment. Patients with concurrent corneal disorders, ocular injury, infection or allergy, and those who wear contact lenses or with follow-up duration of less than two months were excluded from this study.

### Surgical procedures

All procedures were performed by a single surgeon (C.Y.C.) under surgical microscope in the operating room (Video 1). After lid speculum placement and application of topical anesthetic (0.5% proparacaine, Alcaine®, Novartis, NJ, USA), the inferior bulbar conjunctiva was checked for the extent of Cch. Redundant bulbar conjunctiva was grasped with smooth forceps, and a bent electrode tip (OcuRF®, Ilooda, Korea) was inserted into the subconjunctival space. Radiofrequency waves released from the electric unit (0.6 ~ 0.8 watts, 2 MHz, Acutron™, Ilooda, Korea) led to subconjunctival contraction burn via the inserted electrode tip. In most cases, the output power intensity of the radiofrequency unit was set at levels 3–4 (0.6 ~ 0.8 watts, continuous wave) and adjusted intraoperatively depending on the amount of redundant conjunctiva being coagulated without tissue charring. On average, approximately 20 ~ 30 subconjunctival coagulation was performed in the horizontal and vertical directions depending on the shape and amount of conjunctival laxity, and the number of coagulations performed was determined according to the extent of conjunctival redundancy. Care was taken to avoid coagulation of the cornea and the limbus. The power intensity was reduced to 0.4 watts in cases of thin and pliable conjunctiva without sufficient tenon tissue. Once minimal or no redundant tissue was observed, the treated conjunctiva was compressed with wet gauze for 5 s, and 0.5% levofloxacin antibiotic eye drop (Cravit®, Santen, Osaka, Japan) was instilled into the operated eye.

### Outcome measures

All patients were routinely followed up at 2 weeks and 2 months after surgery. Slit-lamp biomicroscopy and anterior slit photography were performed before and after surgery (light illumination: direct intensity − 50%, indirect intensity − 50%, angle 45°). The Cch grade was evaluated after staining the cornea and conjunctiva with 5 µl of 2% fluorescein solution. Improvement in Cch grade was examined by comparing anterior photography before and after surgery. After corneal staining, tear break-up time (TBUT) was measured three times with a stop-watch by a single examiner (C.Y.C.) under the cobalt blue/yellow light filter. Mean values were used for statistical analysis.

All patients with postoperative Cch grade of 0 or 1 were regarded as ‘success’. During the follow-up period, additional treatments were performed when necessary.

The degree of hyperemia in the bulbar and limbal conjunctiva was examined using an Keratograph 5 M (Oculus, Wetzlar, Germany) before and after surgery and scored based on standard photographs from grade 0 to 4. Total, temporal, and nasal values in bulbar and limbal conjunctiva were recorded for analysis. At 2 months after surgery, all eyes were carefully examined for any signs of complications or side effects that may have resulted from the surgery, and patients were inquired for subjective symptoms.

### Postoperative care

All patients received topical 0.1% fluorometholone (Flumetholone®, Santen, Osaka, Japan) and 0.5% levofloxacin (Cravit®, Santen, Osaka, Japan) eye drops three times daily for 2-weeks postoperatively. After two weeks, 0.1% fluorometholone eye drops (Flumetholone®, Santen, Osaka, Japan) were reduced to twice daily until 2 months postoperatively.

### Statistical analysis

Statistical analyses were performed with SPSS software, version 26.0 (SPSS Inc, Chicago, IL). Normality of the data samples was assessed using the Kolmogorov-Smirnov test. Paired *t-test* was used for comparison between pre-and post-operative values of conjunctival hyperemia and TBUT. A *P*-value less than 0.05 was considered statistically significant.

## Results

Medical records of a total of 281 patients who received radiofrequency treatment were analyzed retrospectively. Among these patients, 143 were diagnosed with Cch. A total of 127 patients (230 eyes) with a follow-up period of at least two months were finally included in this study. The mean follow-up period was 11.9 ± 8.6 months and the maximum follow-up period was 25 months. The average age of these patients was 68.0 ± 9.4 years (range, 34 to 89 years) and cataract surgery was the most common past surgical history (Table 1).

**Table 1:** Demographics and clinical characteristics

Variable	*N* (%) or Mean (SD)
Total 230 eyes of 127 patients	
Age, yr *	68.0 (9.4)
Sex*	
Male	40 (31.5%)
Female	87 (68.5%)
Total Follow-up Period (months)	11.9 (8.6)
Preoperative IOP (mmHg)	16.3 (2.4)
Preoperative BCVA (logMAR)	0.1 (0.1)
Medical history	
Dry eye	19 (8.3%)
Glaucoma	1 (0.4%)
Thyroid eye disease	2 (0.9%)
*Surgical history*	
Cataract surgery	80 (34.8%)
Refractive surgery	9 (3.9%)
Blepharoplasty	42 (18.3%)
Intense Pulsed Light treatment	12 (5.2%)
Pterygium surgery	2 (0.9%)
PPV	1 (0.4%)
DCR	1 (0.4%)

*Number of Patients

N, number; SD, standard deviation; Cch, conjunctivochalasis; IOP, intraocular pressure; BCVA, best-corrected visual acuity; logMAR, logarithm of the minimum angle of resolution; PPV, pars plana vitrectomy; DCR, dacryocystorhinostomy

Regarding concurrent ocular comorbidities, 209 (90.9%) eyes were diagnosed solely with Cch, while 7 (3.0%) eyes had chronic chemosis, 2 (0.9%) eyes lymphangiectasis, and 4 eyes (1.7%) conjunctival nevus. Punctal occlusion with radiofrequency was performed in 6 (2.6%) eyes with concurrent tear-deficiency dry eye. Electrolysis of trichiasis was performed in 2 (0.9%) eyes using radiofrequency. All patients received radiofrequency treatment for their comorbidities during the same session as Cch surgery (Table 2).

**Table 2:** Concurrent treatment of ocular comorbidities with radiofrequency (number of eyes, *n* = 230)

	*N* (%)
Cch only	209 (90.9%)
Chemosis	7 (3.0%)
Lymphangiectasis	2 (0.9%)
Conjunctival Nevus	4 (1.7%)
Tear deficiency dry eye	6 (2.6%)
Trichiasis	2 (0.9%)

N, number; Cch, conjunctivochalasis

**Fig. 1:**
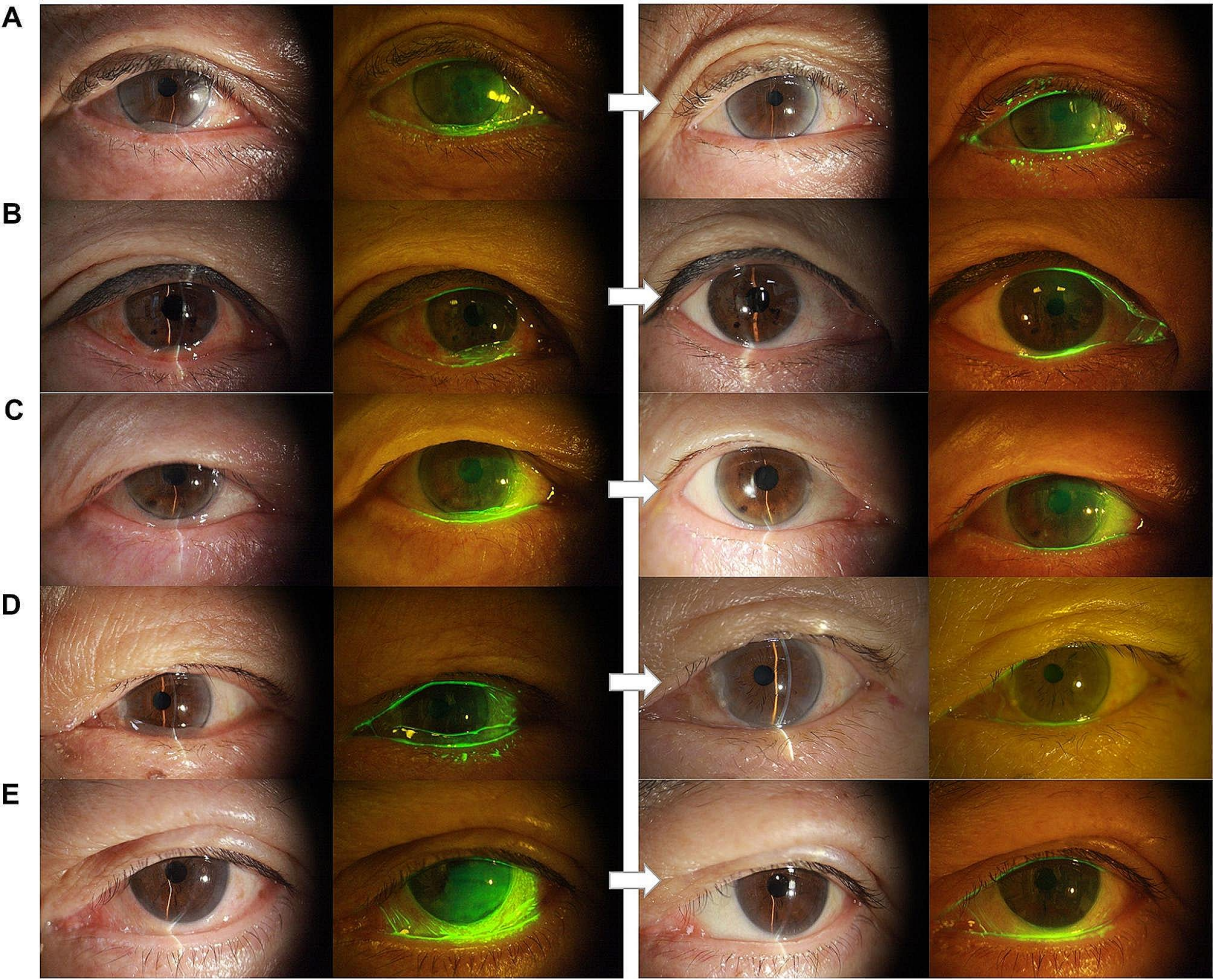
Preoperative and postoperative slit-lamp photographs of the cases (**A**-**E**)(with and without cobalt-blue/yellow filter and fluorescein stain). (**A**-**D**) four cases of Cch in grade 3 preoperatively; (**E**) a case of Cch in grade 2 preoperatively with history of longstanding epiphora for more than 6 months. At 2 months postoperatively, all cases showed grade reduction to 0 or 1

Preoperatively, 165 (71.7%) eyes showed grade 2 Cch and 65 (28.3%) eyes grade 3 Cch. At 2 months postoperatively, 199 (86.5%) eyes and 25 (10.9%) eyes had grade 0 and 1 Cch, respectively (Table 3), yielding 97.4% “success” rate (Fig. 1). In 227 (98.7%) eyes, the Cch grade decreased after surgery.

**Table 3:** Preoperative and postoperative grading of conjunctivochalasis by Meller and Tseng system (number of eyes, *n* = 230)

	Preoperative	Postoperative 2 months
Grade 0	0 (0%)	199 (86.5%)
Grade 1	0 (0%)	25 (10.9%)
Grade 2	165 (71.7%)	3 (1.3%)
Grade 3	65 (28.3%)	3 (1.3%)

A total of 8 (3.5%) eyes received additional treatment, and the mean time interval between the first and second procedures was 3.6 ± 2.5 months (range, 2 month to 9 months). Of 6 eyes (2.6%) that underwent early re-treatment at 2 months postoperatively, three eyes had concurrent chemosis and three eyes were under-corrected during the initial surgery (grade from 3 to 2 in all 3 eyes). Two (0.9%) of 8 eyes received late re-treatment due to recurrence at postoperative 6 months and 9 months, respectively. A total of 2 eyes initially showed treatment “success” from grades 2 and 3 to grade 1 at postoperative 2 months, but recurred to grade 2 later. All 8 eyes improved to grade 0 or 1 with a single additional procedure.

TBUT for 43 eyes improved from 3.17 ± 0.82 s to 5.28 ± 1.10 s after surgery (*P* < 0.001). Conjunctival hyperemia was examined for a total of 30 eyes and all values (bulbar total, temporal, nasal, as well as limbal total, temporal, and nasal) showed statistically significant improvements postoperatively (all *P* values < 0.05) (Table 4). Subconjunctival hemorrhage was observed in 8 (3.5%) eyes during the early postoperative period, which showed spontaneous resolution without additional treatment. Ocular discomfort was the most common subjective complaint during the early postoperative period (28 eyes, 12.2%).

**Table 4:** Postoperative changes of conjunctival hyperemia measured with Keratograph 5 M (*n* = 30 eyes)

	Preoperative, Mean (SD)	Postoperative 2 months, Mean (SD)	*P*
Bulbar			
Total	1.7 (0.6)	1.4 (0.6)	0.012
Temporal	1.8 (0.7)	1.5 (0.7)	0.018
Nasal	1.7 (0.6)	1.4 (0.4)	0.013
Limbal			
Total	1.3 (0.7)	1.0 (0.4)	0.004
Temporal	1.3 (0.7)	1.0 (0.5)	0.003
Nasal	1.2 (0.7)	1.0 (0.4)	0.029

Values are given in mean (standard deviation)

P value, probability value from paired t-test. Statistically significant result with *P* < 0.05

SD, standard deviation

## Discussion

Cch is an entity commonly encountered in daily practice and often misdiagnosed as an ocular surface disease, causing several symptoms similar to dry eye [10, 15]. Several theories exist on the pathophysiology of Cch. Age-related conjunctival laxity combined with gradual disintegration of Tenon’s capsule, which can weaken the adhesion between the conjunctiva and the sclera, may give rise to Cch [5, 16]. Continuous mechanical friction between the lower lid and the conjunctiva may also obstruct the lymphatic flow, predisposing Cch formation [3]. Histopathologically, Cch is attributed to elastosis, fragmentation of elastic fibers, and loss of collagen [15, 17]. Increases in inflammatory cytokines, IL-1 and IL-6, and proteins such as matrix metalloproteinase (MMP) have also been shown to be associated with Cch as they promote collagenolytic activity in conjunctival cells [18].

Several surgical approaches exist for symptomatic Cch recalcitrant to medical treatment, such as surgical excision, conjunctival cauterization, scleral fixation suture, conjunctivoplasty with or without amniotic membrane, and conjunctivoplasty with argon laser. In conventional surgical excision, longer operation time and postoperative complications such as pain, discomfort, abscess formation, suture-related pyogenic granuloma formation, giant papillary conjunctivitis, ocular motility restriction, and infection should be considered [1, 16, 19]. In addition, there is a risk of over- or under-resection depending on the amount of excision based on the intraoperative judgment of the surgeons [10]. In one report, 34.8% of Cch persisted after surgery due to incomplete resection [20]. In conjunctival cauterization, excessive conjunctival contraction, adhesion with subconjunctival tissues, postoperative pain and discomfort, subconjunctival hemorrhage, scarring, and symblepharon formation may occur [10, 21, 22].

Our previous studies have demonstrated that the use of radiofrequency is an excellent and effective method for reducing Cch with less thermal damage [11, 23]. However, in those studies, we used a radiofrequency device that features a wide electrode tip width of 120 microns even at its thinnest point. This is because such radiofrequency units were mainly used in general surgery during that time with an energy output range of 0 to 120 watts and there were no electrodes available that were specifically designed for microsurgical ophthalmic purposes. The new radiofrequency unit used in the present study comes with an electrode with 68-micron shaft thickness and tip width of a mere micron, which allows an extremely precise and controlled treatment of thin and delicate structures such as ocular conjunctiva with minimal risk of scleral perforation or limbal stem cell damage. Furthermore, it generates radiofrequency energy between 0.2 and 2 watts (adjustment unit: 0.2 watts per each level), which is ideal and sufficient for the treatment of conjunctival diseases.

The general principle of radiofrequency treatment is that when the high frequency is applied to the targeted tissue through the electrode tip, intracellular water boiling and coagulation occur [10]. Cellular volatilization is a phenomenon in which cells are destroyed by an increase in intracellular pressure, resulting in the contraction of the relaxed and degenerated conjunctiva tissue [23]. Cch treatments using high frequency are advantageous in that they have less scarring by reducing tissue damage with less heat, faster healing, no suture-related complications, and less discomfort after surgery [10, 12, 14].

In this study, a single radiofrequency treatment led to treatment success in 97.4% of cases which is higher than the success rate observed in our previous study where 95% (19 out of 20 eyes) showed improvement of Cch to grade 0 or 1 after radiofrequency treatment [11]. Considering that 50% and 20% of the patient population in the previous study showed pre-operative Cch grades of 2 and 3^11^, respectively, compared to 71.2% and 28.3% in the present study, our results suggest that utilization of ultra-thin electrode specifically designed for ocular conjunctival indications with delicate and precise energy control can lead to more effective Cch treatment.

Only 3.5% (8 of 230 eyes) required an additional treatment in this analysis, which is comparable to 5% (1 of 20 eyes) re-treatment rate reported in our previous study [11]. All eyes that underwent re-treatment showed improvement to Cch grade 0 or 1 after a single additional treatment. Among those that required additional treatment, 2.6% (6 of 230 eyes) received early re-treatment at postoperative 2 months due to concurrent chemosis and under-correction during the initial procedure, while 0.9% (2 of 230 eyes) underwent late re-treatment due to recurrence. This suggests that the risk factors of early re-treatment may be the comorbidity with chemosis or under-correction and that of late re-treatment may be the recurrence.

Higher Cch grade is known to be associated with shorter TBUT and stronger conjunctival friction, which can result in disruption of tear dynamics and degradation of ocular surface quality [13]. Another study demonstrated that a decrease of mucin level in Cch may also negatively affect TBUT and the ocular surface [24]. In the present study, we observed a post-surgical increase in TBUT and reduction in ocular redness, suggesting that the radiofrequency treatment of Cch can lead to overall improvement of tear film dynamics and ocular surface quality.

Regarding postoperative complications, ocular discomfort was the most common, observed in 28 (12.2%) of 230 eyes. Conjunctival injection lasting longer than 2 weeks was observed in 17 eyes (7.4%), while 18 (7.8%) eyes showed subconjunctival hemorrhages. However, all postoperative complications resolved spontaneously within two months. Most importantly, in all treated eyes, no serious complications such as conjunctival fibrotic scar formation, thinning, avascular change, scleral melting, symblepharon, or wound dehiscence occurred. The complication rates observed herein are similar to those observed in previous studies which also used radiofrequency waves for Cch treatment [11, 12].

The average age of the 127 patients included in this study was 68.0 ± 9.4 years. Previous studies have reported that Cch occurs in 98% of people over the age of 60^2,24^, and that the prevalence of Cch increases with age [2, 25, 26]. In contrast to a previous report on clinical characteristics of patients with Cch, our study demonstrated a strong female predominance (87 women and 40 men) which suggests that Cch may be more prevalent among females in Korean population [15].

This study is not without limitations. Due to the relatively short mean follow-up period of 11.9 months, future studies with a longer follow-up time are necessary to confirm the long-term clinical outcomes and complication rates observed in this study. Pain score analysis was not conducted as all patients reported insignificant pain, described as foreign body sensation or mild stinging during and after the procedure. Future studies should incorporate pain analysis.

Nevertheless, our results suggest that Cch treatment using new ophthalmic radiofrequency device with ultra-thin electrode reduced the Cch grade with high success rate and no serious complications. The treatment also led to improvements in TBUT and conjunctival redness. We also showed treatment courses in eyes with concurrent comorbidities which were not described in previous studies. Even in cases of early or late re-treatment, the treatment outcomes were improved with a single additional procedure. Thus, ophthalmic radiofrequency may be considered a favorable first-line therapeutic approach for Cch.

### Electronic supplementary material

Below is the link to the electronic supplementary material.

Supplementary Material 1

Supplementary Material 2

## Data Availability

No datasets were generated or analysed during the current study.
