# Supplementary material for: Clinical outcomes of conjunctivochalasis treatment with a new ophthalmic radiofrequency device
Source: BMC Ophthalmol. 2024 Jul 22;24:302. doi: 10.1186/s12886-024-03499-2 (PMC11265150; doi:10.1186/s12886-024-03499-2)
Supplement: Supplementary file 2 — Supplementary Material 2 [file 12886_2024_3499_MOESM2_ESM.docx]

Video 1. Conjunctivochalasis surgery using a new ophthalmic radiofrequency device.
